# New Protocol for Quantitative Analysis of Brain Cortex Electroencephalographic Activity in Patients With Psychiatric Disorders

**DOI:** 10.3389/fninf.2018.00027

**Published:** 2018-05-24

**Authors:** Grzegorz M. Wojcik, Jolanta Masiak, Andrzej Kawiak, Piotr Schneider, Lukasz Kwasniewicz, Nikodem Polak, Anna Gajos-Balinska

**Affiliations:** ^1^Faculty of Mathematics, Physics and Computer Science, Institute of Computer Science–Department of Neuroinformatics, Maria Curie-Sklodowska University in Lublin, Lublin, Poland; ^2^Neurophysiological Independent Unit of the Department of Psychiatry, Medical University of Lublin, Lublin, Poland

**Keywords:** electroencephalography, sLORETA, psychiatric disorders, quantitative analysis, biomarkers, P300

## Abstract

The interview is still the main and most important tool in psychiatrist's work. The neuroimaging methods such as CT or MRI are widely used in other fields of medicine, for instance neurology. However, psychiatry lacks effective quantitative methods to support of diagnosis. A novel neuroinformatic approach to help clinical patients by means of electroencephalographic technology in order to build foundations for finding neurophysiological biomarkers of psychiatric disorders is proposed. A cohort of 30 right-handed patients (21 males, 9 females) with psychiatric disorders (mainly with panic and anxiety disorder, Asperger syndrome as well as with phobic anxiety disorders, schizophrenia, bipolar affective disorder, obsessive-compulsive disorder, nonorganic hypersomnia, and moderate depressive episode) were examined using the dense array EEG amplifier in the P300 experiment. The results were compared with the control group of 30 healthy, right-handed male volunteers. The quantitative analysis of cortical activity was conducted using the sLORETA source localization algorithm. The most active Brodmann Areas were pointed out and a new quantitative observable of electrical charge flowing through the selected Brodmann Area is proposed. The precise methodology and research protocol for collecting EEG data as well as the roadmap of future investigations in this area are presented. The essential result of this study is the idea proven by the initial results of our experiments that it is possible to determine quantitatively biomarkers of particular psychiatric disorders in order to support the process of diagnosis and hopefully choose most appropriate medical treatment later.

## 1. Introduction

In the last two decades renaissance of electroencephalographic techniques was observed. Known for more than one hundred years the electroencephalography (EEG) was mainly used for the diagnosis of epilepsy. Recently there has been a rapid expansion of Brain-Computer Interfaces (BCI) in which the acquisition of electrical activity of selected areas of brain cortex plays the main role (Mikolajewska and Mikolajewski, 2012, 2013, 2014) as in all the works related to Steady State Visually Evoked Potentials (SSVEP) (Kotyra and Wojcik, 2017b) or neurofeedback largely related to it (Lubar et al., 1995; Kotyra and Wojcik, 2017a) or other cognitive research (Martínez-Rodrigo et al., 2017). New neuroimaging methods such as Low-Resolution Brain Electromagnetic Tomography (LORETA) (Pascual-Marqui et al., 1994) and its standardized version (sLORETA) (Pascual-Marqui, 2002) were developed. Owing to these new quantitative and visualization methods brain activity can be investigated in new aspects. In addition, using EEG is cheaper than using Magnetic Resonance Imaging (MRI), noninvasive and has much better temporal resolution compared to the in functional MRI (Tohka and Ruotsalainen, 2012).

The clinical interview is still the main diagnostic method used in current psychiatry. However, the EEG seems to be a useful tool to support the psychiatrist (Sand et al., 2013). A wide range of psychiatric disorders is not possible to be diagnosed based on MRI, while some EEG methods seem to be appropriate for the diagnosis support in psychiatry (Kamarajan and Porjesz, 2015).

Our investigations were aimed at finding whether there are some biomarkers in the electrical cortical activity of the brain and whether they are characteristic of particular disorders as in some attempts made by John in late eighties (John et al., 1988). Some neurophysiological markers were found for example in research on burn-out syndrome (Golonka et al., 2017) which is also in the area of our interests (Chow et al., 2018).

Event-Related Potentials (ERP) were extensively investigated by experimental psychologists (Campanella, 2013), among others, to understand better engagement and working memory mechanisms (Pope et al., 1995; Chaouachi et al., 2010; Jones and Macken, 2015).

Cognitive functions in the patients with psychiatric disorders are not as effective as among healthy representatives of the populations (Niedermeyer and da Silva, 2005; Trivedi, 2006). Decision-take processes and reaction time seem to be crucial in these areas and our own studies as well as literature reviews (Brown et al., 1982) show that there were many attempts to carry out research on patients with disorders using P300. Some P300 analyses of patients with schizophrenia are presented in Jeon and Polich (2003) while panic disorders were investigated, e.g., in Clark et al. (1996). Phobias, among others spider phobia manifesting in P300 waves are presented in Leutgeb et al. (2009), Scharmüller et al. (2011), and Kolassa et al. (2006). The amplitude of P300 was investigated by Gangadhar in the early nineties (Gangadhar et al., 1993) for melancholic non-bipolar depression and by Himani in major depression (Himani et al., 1999). The auditory version of P300 is quite often used for the investigations of patients with Autism Spectrum Disorder (Souza et al., 1995; Devoto et al., 2003; Schulze et al., 2008; Cui et al., 2017) whereas the P300 for sleep disorders in Devoto et al. (2003). That is why we decided to use P300 for the research carried out on our cohort including the patients with all above mentioned diagnoses.

The paper presents the quantitative analysis of brain cortex electrical activity using the sLORETA algorithm for the signal collected by the dense array EEG amplifier supported by a photogrammetric station from the patients with selected psychiatric disorders compared to the control group. The research protocol using ERP in patients is also presented. A new observable that will allow to assess how much electrical charge flew through the particular Brodmann Area (BA) is introduced.

## 2. Materials and methods

It is assured that many EEG biomarkers can manifest themselves during the decision-making process and that is why the Event-Related Potentials (ERP) experiments ought to be suitable for their detection. There was chosen the well-known P300 experiment (Chapman and Bragdon, 1964; Sutton et al., 1965) to investigate a group of patients with a wide range of psychiatric disorders classified in ICD-10 as: F20 (schizophrenia), F31 (bipolar affective disorder), F32.1 (moderate depressive episode), F40 (phobic anxiety disorders), F41 (other anxiety disorders, panic disorder), F42 (obsessive-compulsive disorder), F51.1 (nonorganic hypersomnia), F84.5 (Asperger syndrome).

There are several procedures to evoke the P300 wave. In general, the idea of P300 experiment is strongly associated with the methodology of Event-Related Potentials (Nidal and Malik, 2014). The subject is most often asked to press a button whenever he or she can see the awaited symbol (called “Target”) on the monitor screen. There are two kinds of symbols appearing on the screen. Thus besides Targets (TGTs), there are the symbols called “Standard” (STDs). In P300 there are always much fewer TGTs than STDs and their proportion in the whole set of symbols is to be set. The ERP wave has statistical characteristics so many trials must be made to calculate the wave for each subject precisely. STDs and TGTs are usually shown in the series in the number from several dozens to several hundred symbols shown in each series.

Both kinds of symbols were shown on black screens for 500 ms. To sum up, the activity during the cortical response of the subject to 300 symbols (3 series × 100), with the total 60 TGTs and 240 STDs was collected.

During the experiment patients were asked to press a button on the response pad whenever they saw the “cross” sign on the monitor screen. There were four series (first of them was a testing series) of 100 stimuli in each, 20% of stimuli were white crosses as TGTs and 80% were white circles as STDs. In all subjects, the P300 wave appeared, however, it was the most important for us to check which region of the brain was active in the case of particular subject compared to the control group with the distinction of TGT and STD responses.

**Figure 1:**
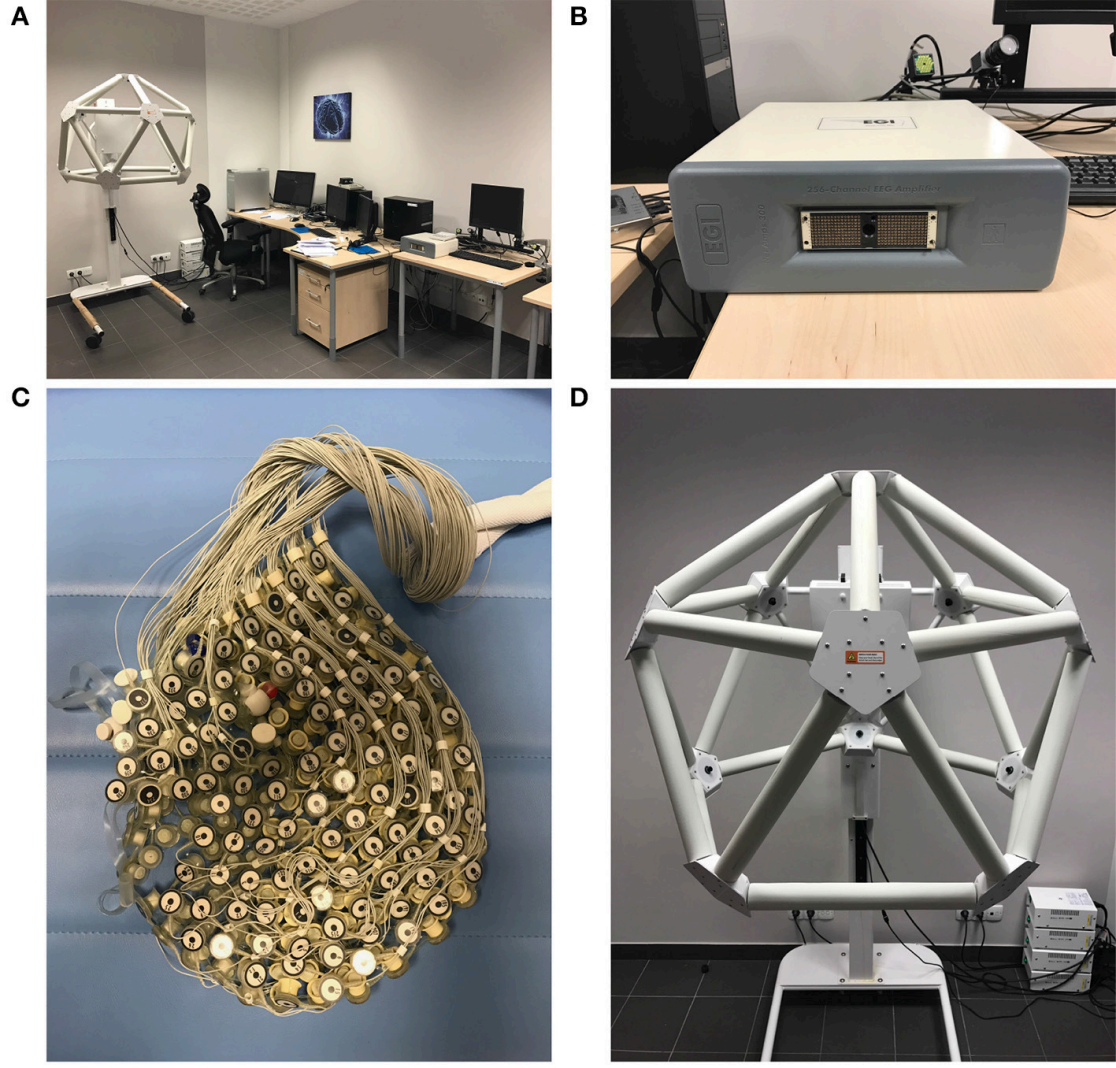
EEG Laboratory in the Department of Neuroinfomatics. From top-left corner clockwise: **(A)** general view of the lab, **(B)** 256 channel EEG dense array amplifier, **(D)** GPS station equipped with 11 cameras for the GeoSource software, and **(C)** 256 electrodes HydroCel GSN 130 Geodesic Sensor Net.

In the Department of Neuroinformatics there was 256-channel dense array EEG amplifier provided by EGI^1^ with the Net Station v. 4.5.4 signal acquisition software. Laboratory (see Figure 1) is also supported by the Geodesic Photogrammetry System (GPS) with the Net Local 1.00.00 and GeoSource 2.0 software that are able to conduct the source localization procedure and sLORETA visualization. Saccadic eye movements and eye blinks were eliminated by the SmartEye 5.9.7 controlling the eye-tracker system that is an integral part of EGI lab. Event-Related Potentials (ERP) experiments were prepared in the PST e-Prime 2.0.8.90 tool^2^.

**Figure 2:**
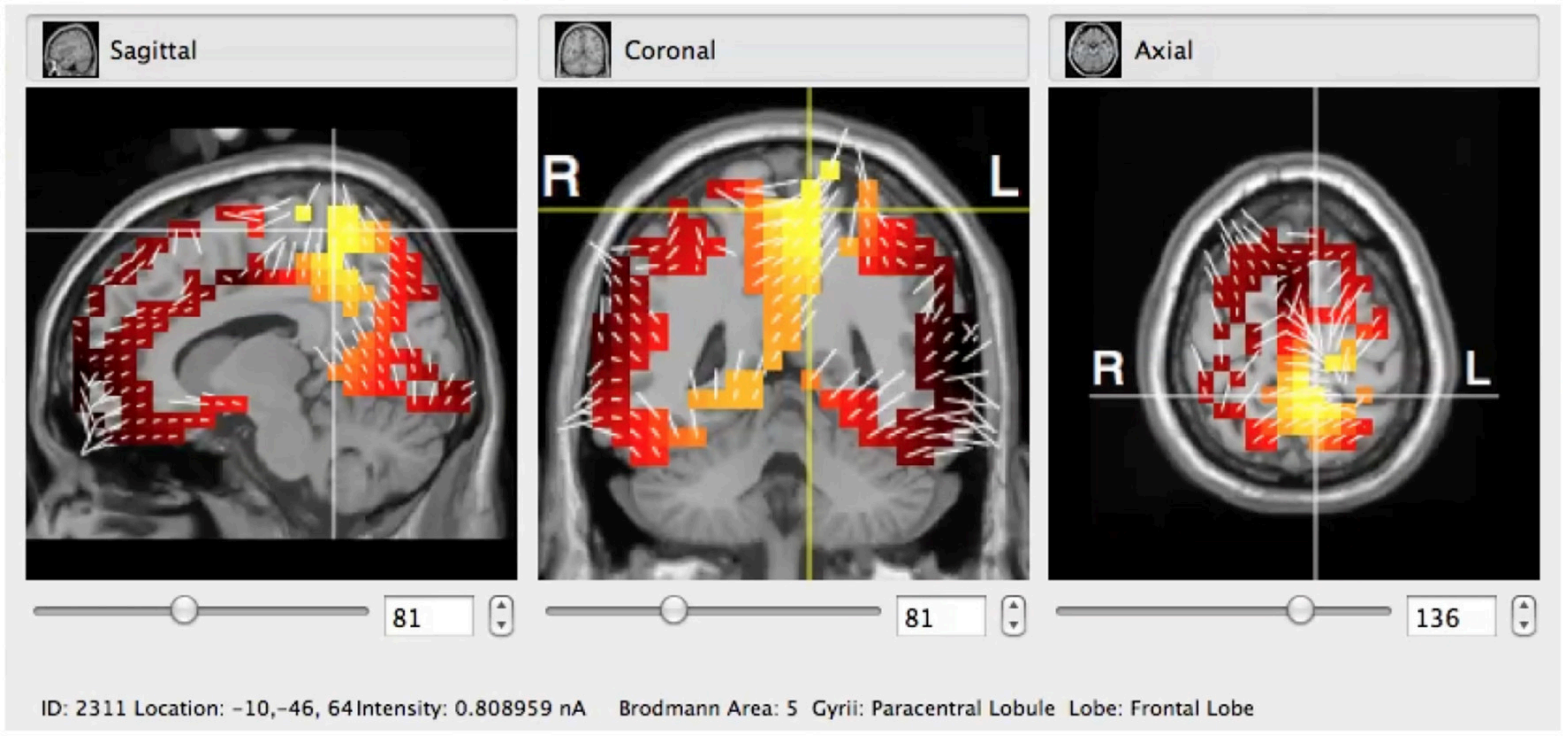
Typical visualization of the sLORETA algorithm applied to the GeoSource pre-processed raw EEG signal in coronal, sagittal, and axial cross-sections. Here the BA5 (Paracentral Lobule, Frontal Lobe) is indicated.

After the EEG signal was collected the pre-processing analysis was made by removing artifacts (mainly eye-blinking and saccades) and later there was applied sLORETA algorithm to it (Figure 2). Due to the appliance of GPS it was possible to indicate with a very good precision to the subject's cortex BAs that were the most active during his or her responses to the STD and TGT stimuli. The time interval in which the BA activity was calculated was set to 5 ms.

The sLORETA used in our Laboratory was the most standard version of the algorithm described in detail in the Brain Source Localization Using EEG Signals chapter of Nidal and Malik (2014). The sLORETA method assumes the standardization of the current density. That means that not only the variance of the noise in the EEG measured signal is taken into account but also that the biological variance in the actual signal is considered (Goldenholz et al., 2009; Nidal and Malik, 2014). These biological activity changes are taken as independently and uniformly distributed across the brain. This results in a linear imaging localization technique having an exact zero-localization error (Goldenholz et al., 2009; Nidal and Malik, 2014). The perfect and detailed comparison of different variations of LORETA is presented in Nidal and Malik (2014).

**Figure 3:**
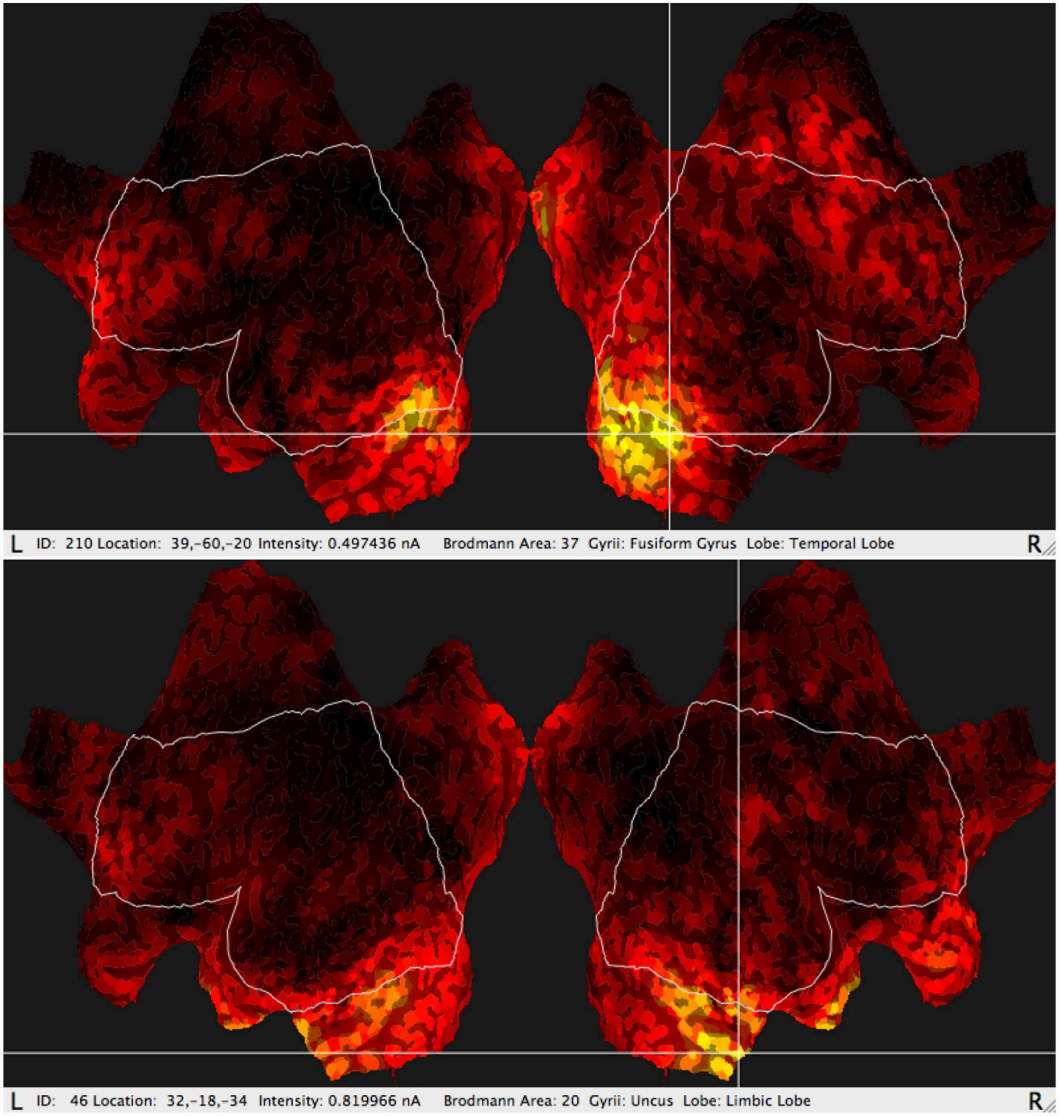
Typical results of GeoSource BA activity visualization on the brain cortex so-called Flat Map. The increase of activity in BA37 (Fusiform Gyrus, Temporal Lobe) and BA20 (Uncus Lobe, Limbic Lobe) is indicated.

In addition, the GeoSource software makes it possible to estimate amperage of the most active areas (Figure 3) varying in time. The reason for which a new observable was considered originated from the fact that particular BA could last at its maximum value for a longer or shorter period of time and it could appear more than once during each epoch. The signal was divided into epochs, as usual in P300 experiments, then averaged giving amperage in function of time.

The electric current flowing through each BA is expressed by:

(1) $$I\left(BA,\tau ,t,|\Psi \rangle \right)=\frac{\partial q(BA,\tau ,t,|\Psi \rangle )}{\partial t}$$

where *q*(*BA*, τ, *t*, |ψ〉) is the electric charge which accumulates in the BA for estimated period time after the stimulation τ and surely depends on some set of psychophysiological parameters represented by the vector |Ψ〉.

Our new observable ι (Iota) is defined as the electric charge that flew through the given BA given by the integral:

(2) $$\forall BA:\iota =q\left(BA,\tau ,t,|\Psi \rangle \right)=\int_{\tau +t_{1}}^{\tau +t_{2}}I\left(BA,\tau ,t,|\Psi \rangle \right)dt$$

in the time range limited by *t*_1_ = 280 ms and *t*_2_ = 600 ms after stimulation that took place in time τ. That range is reasonably chosen in P300 investigations as the most appropriate time to observe P300 wave.

Based on the electrical current measured by the amplifier, particular BAs precisely indicated by the photogrammetry station and having sharply estimated time intervals owing to the perfect EEG time resolution, one of many numerical methods for integration can be applied to calculate ι with good precision. This variable can be calculated for each BA, for everyone, no matter if the subject is healthy or suffers from any disorder. However, the systematic clinical validation must be conducted and finding correlations with the clinical symptoms as well as the comparison between healthy peers should be of top priority in future research. The aim of this paper was not to solve the above mentioned medical problems but to show the method that may be useful for working out new diagnosis support tools.

**Figure 4:**
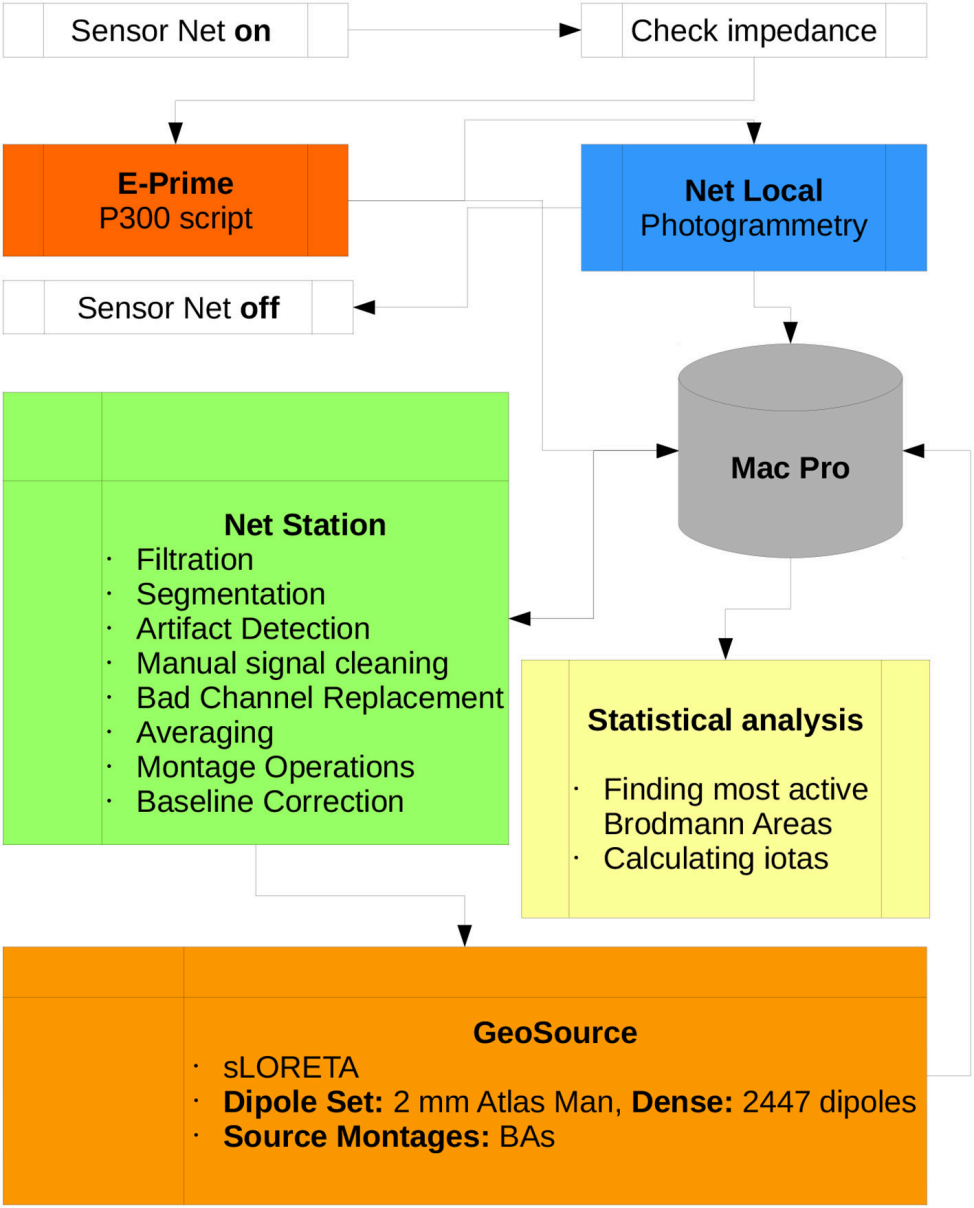
Diagram of the P300 research protocol proposed in this paper. Participation of the subject in the experiment begins when the Sensor Net is put on and ends when it is taken off. All data is collected by the Mac Pro workstation which is the central part of the lab. All scripts used for preprocessing data in Net Station and postprocessing in GeoSource are listed. Statistical analysis, finding the most active BAs and calculating ι for each of them can be conducted using other machines.

The Scheme of the methodology and research protocol are presented in Figure 4.

## 3. Results

The cohort of untreated 30 patients, 21 right-handed males, and 9 right-handed females (avg. age 28.1, s.d. 12.4) was investigated in the P300 experiment. All patients participated in the experiment before taking the first dose of suggested medications.

The patients were with a wide range of diagnosed psychiatric disorders classified in ICD-10 as: 1 × F20 (schizophrenia), 3 × F31 (bipolar affective disorder), 4 × F32.1 (moderate depressive episode), 2 × F40 (phobic anxiety disorders), 12 × F41 (other anxiety disorders, panic disorder), 2 × F42 (obsessive-compulsive disorder—among patients with F84.5), 2 × F51.1 (nonorganic hypersomnia), 6 × F84.5 (asperger syndrome).

The results are compared with the participants from the control group of 30 healthy volunteers, males (avg. age 22.4, s.d. 1.7)^3^.

The members of the control group were right-handed (like all the subjects) males and claimed that they had neither psychiatric no neurological problems. They were not treated before. They confirmed not taking medicines in a regular way. They were not drug addicted and did not use alcohol for at least two days prior the investigations. The control group was selected from the students of Computer Science and Cognitive Science at Maria Curie-Sklodowska University in Lublin, Poland. All of them estimated their general medical condition as good or very good. Only the subjects with the above characteristics could become members of the control group. Females, left-handed and all with neurological traits were excluded in this stage of the project. We are aware of the fact that gender plays a significant role in EEG experiments. As a baseline, there were chosen only males for the control group and in future, separate research for particular genders is going to be carried out.

The procedure of estimating the most active BAs was as follows: after the signal acquisition, the photo of the subject was taken using 11 cameras in the GPS. Then there was calculated the activity of particular BAs (in nanoamperes) varying in time and this varying activity together with its corresponding BAa was saved in the appropriate list using the GeoSource software. Then the scripts chose the activity that was the greatest in a given interval of time. Not only the maximum value of the electrical current of given BA in a given interval was considered but also the time in the range of this interval in which this activity was maintained. In other words, the maximum activity was equivalent to the electric charge that flew through the given area.

The Results for the group of patients are presented in Table 1 (most active BA) and Table 2 (ι calculated for the most active BA).

**Table 1:** Most active BA in particular patients during the P300 experiment as obtained from the sLORETA quantitative analysis.

**Patient No**.	**Diag**.	**STD most active BA**	**TGT most active BA**
1	F20	L23	S1
2	F31	R46	R46
3	F31	S1	S1
4	F31	R9	R9
5	F32.1	S1	R9
6	F32.1	LHipp, R41	LHipp, R41
7	F32.1	L36, R36	L36, R36
8	F32.1	R9	R36
9	F40	R41	LAmyg
10	F40	R46, R41	R46, R42
11	F41	S1	R44, L43
12	F41	S1	R9
13	F41	S1	S1
14	F41	R28	S1
15	F41	S1	S1
16	F41	R9	R9
17	F41	S1	S1
18	F41	L27, L33	L27
19	F41	S1	L45
20	F41	R4	LAmyg
21	F41	S1	R45
22	F41	S1	S1
23	F51.1	L36, R9	L27
24	F51.1	S1	S1
25	F84.5	L45	LHipp
26	F84.5	Amyg, R44	R9
27	F84.5, F42	L45	L45
28	F84.5, F42	R9	R9
29	F84.5	L45, R9	R9
30	F84.5	R39, LHipp	L37, L45

*“Amyg” stands for Amygdala, “Hipp” for left Hippocampus areas. S1 for the areas of Primary Somatosensory Cortex. L-R for Left - Right hemisphere, respectively*.

**Table 2:** The ι for the most active BA in particular patients during the P300 experiment for the STD and TGT responses obtained from the sLORETA quantitative analysis.

**No**.	**Diag**.	**ι (STD)[μC]**	**ι (TGT) [μC]**
1	F20	33.5	S1
2	F31	2.11	20.1
3	F31	S1	S1
4	F31	76.4	75.3
5	F32.1	S1	75.5
6	F32.1	5.20, 2.35	2.99, 28.5
7	F32.1	17.3, 21.0	24.4, 14.4
8	F32.1	5.68	S1
9	F40	1.07	2.09
10	F40	10.6, 5.66	38.9, 16.1
11	F41	S1	139, 629
12	F41	S1	2.53
13	F41	S1	S1
14	F41	3.44	S1
15	F41	S1	S1
16	F41	3.29	25.3
17	F41	S1	S1
18	F41	13.4, 13.3	43.7
19	F41	S1	3.72
20	F41	0.855	1.85
21	F41	S1	20.5
22	F41	S1	S1
23	F51.1	18.2, 21.4	63.8
24	F51.10	S1	S1
25	F84.5	3.98	2.17
26	F84.5	1.25, 1.75	6.21
27	F84.5. F42	38.7	124
28	F84.5. F42	1.7	4.63
29	F84.5	13.3, 1.92	30.3
30	F84.5	23.8, 5.88	78.5, 6.48

*S1 indicated the activity in Primary Somatosensory Cortex and was neglected in the calculations of ι. Compare with Table 1*.

The Results for the control group are presented in Table 3 (most active BA) and Table 4 (ι calculated for most active BA).

**Table 3:** Most active BA in the particular subjects of the control group during the P300 experiment as obtained from the sLORETA quantitative analysis.

**No**.	**STD most active BA**	**TGT most active BA**
1	S1	L33, L9
2	S1	S1
3	S1	L46
4	S1	R9
5	L18	S1
6	R9	S1
7	R9	S1
8	R33	R33, R45
9	R23	R45
10	RHipp	LHipp, R9
11	S1	R9
12	S1	R9
13	R9	R41, L33
14	R7	S1
15	R9	S1
16	R33	R33
17	S1	R9
18	L44, L33	R44
19	S1	S1
20	S1	S1
21	S1	S1
22	S1	S1
23	R9	R9, L46
24	S1	L45
25	LAmyg, R33	S1
26	R9	L46
27	R9	R9
28	R9	R9
29	S1	LAmyg
30	S1	S1

*“Amyg” indicates Amygdala, “Hipp” the Hippocampus areas. S1 for the areas of Primary Somatosensory Cortex. L-R for the Left-Right hemisphere, respectively*.

**Table 4:** The ι for the most active BA in particular subjects of the control group during the P300 experiment for the STD and TGT responses obtained from the sLORETA quantitative analysis.

**No**.	**ι (STD) [μC]**	**ι (TGT) [μC]**
1	S1	4.46.4.14
2	S1	S1
3	S1	8.31
4	S1	3.12
5	2.5	S1
6	2.5	S1
7	3.75	S1
8	11	29.2;8.90
9	9.47	11
10	4.33	4.61;52.8
11	S1	55.4
12	S1	70.5
13	19.9	20.9; 2.54
14	1.64	S1
15	1.04	S1
16	18.7	55.6
17	S1	11.9
18	8.65; 24.9	24.9
19	S1	S1
20	S1	S1
21	S1	S1
22	S1	S1
23	28.5	8.49;1.78
24	S1	19.2
25	7.66;1.12	S1
26	6.95	1.71
27	5.29	3.26
28	54.9	3.2
29	S1	1.91
30	S1	S1

*S1 indicates the activity in Primary Somatosensory Cortex and was neglected in the calculations of ι. Compare with Table 3*.

Determining biomarkers for different psychiatric patients that have very different symptoms and clinical characteristics seem a challenging task. The aim of this paper was not, however, to hypothesize dysfunctions of some parts of the brain in particular disorders but to show a new way in which this can be done. In the group of 30 there were representatives of 8 different diagnoses. Under ideal conditions it would be proper to have c.a. 30 patients of each gender and handedness as well as in three ranges of age. That would make us to record systematically the electrical activity of 1,440 patients only for these 8 disorders. However, there are much more described in ICD-10.

Most active BAs were manually counted for particular disorders reported in Table 1. It is still too far to define any psychiatric hypotheses, however, in the next section, the most active BAs together with their known function in the brain are presented.

## 4. Discussion

In all participants both from the patients and control groups high activity of Primary Somatosensory Cortex was observed. BA1, BA2, and BA3 are all together marked as S1 in Tables 1, 3. It is not surprising as all subjects used a response pad to press the button each time they saw TGT. Nevertheless, it can be interesting that S1 was active even in lots of STD trials when fingers were on the response pad but they were not clicking.

There is no clear difference between the subjects from the patients and the control group that would allow us to show biomarkers characteristic of particular disorders.

However, in the group of patients suffering from F41 (12 patients), both left and right BA45 and right BA44 were hyperactive in a few TGT cases. Anatomically BA45 and BA44 are in the Broca's areas and are supposed to play a role in semantic tasks (Buckner, 1996; Gabrieli et al., 1998). This activity was not expected in the standard P300 experiment.

Among the patients suffering from F32.1, both left and right BA36 (perirhinal cortex in the rhinal sulcus) seem to be more active than anywhere else. According to Murray (Murray et al., 2007), this region is involved in perception and memory.

For the patients with the Asperger syndrome (F84.5), a larger activity can be found in the left BA45 as well as in Hippocampus.

This is the initial stage of our project. The research protocol is proposed and it is hypothesized that there are characteristic biomarkers in the EEG signal for selected disorders. The is also introduced the measure called ι that may be helpful in some cases to enrich typical source localization based quantitative analysis.

Such an approach can be treated as a foundation of new methods for support of diagnosis in psychiatry. Finding neurophysiological biomarkers of psychiatric disorders can be easier if the results from Tables 2, 4 are compared with iotas from Tables 2, 4. However, to achieve any clinical results, much more patients that were not treated before are needed. With so many disorders listed in ICD-10, this task seems to be extremely hard but not impossible. This will require the design of appropriate neuroinformatic infrastructure, e.g., like Bigdely-Shamlo et al. (2016).

The method shown herein will require definitely some improvements and support. Probably the analysis using the artificial neural networks that have lots of applications in medical problems (Szaleniec et al., 2008, 2013) could be also helpful for predictions and qualification of psychiatric disorders (Cavanagh et al., 2017). On the other hand, our hypotheses could be supported by using modeling of neural activity in which we are experienced (Wojcik et al., 2007; Wojcik and Kaminski, 2008; Wojcik and Garcia-Lazaro, 2010). Good modeling can shed some light on the ERP design (Ważny and Wojcik, 2014; Wojcik and Ważny, 2015) especially when electrophysiological parameters of neural cells are taken into account (Wojcik and Kaminski, 2007; Wojcik, 2012). It is certain, that joining skills from a wide spectrum of neuroscience will at last lead to new discoveries and better understanding of the human brain, behavior, and mental dysfunctions. Moreover, better understanding will allow designing new tools to help human beings.

## Ethics statement

This study was carried out in accordance with the recommendations of Guidelines for Good Clinical Practice (GCP). The protocol was approved by the Medical University of Lublin Bioethical Commission. All subjects gave written informed consent in accordance with the GCP. Permission No. KE-0254/140/2015 given by Medical University of Lublin Bioethical Commission on May 28th, 2015.

## Author contributions

GW: project idea and coordination, experiment design, subjects' recruitment, results' interpretation. JM: project idea, experiment design, subjects' recruitment, psychiatric diagnosis, results' interpretation. AK: work in laboratory, cleaning signal, computations, statistical analysis. PS and LK: statistical analysis, writing scripts, work in laboratory, cleaning signal. NP: work in laboratory. AG-B: work in laboratory.

### Conflict of interest statement

The authors declare that the research was conducted in the absence of any commercial or financial relationships that could be construed as a potential conflict of interest.

## Footnotes

^1^Electrical Geodesic Systems, Inc., 500 East 4th Ave. Suite 200, Eugene, OR 97401, USA.

^2^Psychology Software Tools, Inc.PST, Sharpsburg Business Park, 311 23rd Street Ext., Suite 200, Sharpsburg, PA 15215-2821 USA.

^3^In fact, there were investigated about 30% more subjects both patients and control group, but all those for whom the signal was too noisy or incomplete were eliminated.
